# Antimicrobial stewardship in hospitals: effective and indispensable, yet structurally fragile

**DOI:** 10.3389/fpubh.2026.1804970

**Published:** 2026-06-26

**Authors:** Esteban Zavaleta-Monestel, Rochele Mosmann-Menezes, Jeaustin Mora-Jiménez, Sebastián Arguedas-Chacón, Jose Pablo Diaz-Madriz

**Affiliations:** 1 Health Research Department, Hospital Clinica Biblica, San Jose, Costa Rica; 2 Antimicrobial Stewardship Program, Clinical Pharmacy Service, Hospital Santa Cruz, Santa Cruz do Sul, Brazil; 3 Antimicrobial Stewardship Program, Clinical Pharmacy, Hospital Clinica Biblica, San Jose, Costa Rica

**Keywords:** antimicrobial resistance, antimicrobial stewardship, health system governance, hospital governance, quality of care

Antimicrobial resistance (AMR) is no longer a distant or theoretical threat. It is a daily reality in hospitals, where clinicians increasingly confront infections that are more difficult to treat, slower to resolve, and associated with higher morbidity and mortality. Acute care settings concentrate many of the drivers of resistance, including high antimicrobial use, invasive procedures, clinical complexity, and persistent diagnostic uncertainty. Hospitals therefore occupy a central position in both the emergence of AMR and the response to it. A large global analysis estimated that, in 2019, bacterial antimicrobial resistance was associated with nearly five million deaths worldwide, with more than one million deaths directly attributable to AMR, placing it among the leading causes of death globally (1).

This opinion article argues that the persistent fragility of hospital antimicrobial stewardship programs is not the result of insufficient evidence, professional resistance, or technical failures of implementation, but rather of structural and governance choices that frame stewardship as a discretionary activity instead of essential patient safety infrastructure. By examining stewardship through a structural lens, this article contends that its institutional vulnerability is largely produced and perpetuated by the organizational arrangements of health systems themselves.

Over the past two decades, antimicrobial stewardship programs have become a cornerstone of hospital strategies to address AMR. Few interventions in infectious diseases are supported by such a consistent and mature evidence base. Stewardship improves the appropriateness of antimicrobial therapy, reduces unnecessary exposure, and lowers rates of *Clostridioides difficile* infection and antimicrobial-resistant organisms, without compromising patient outcomes. In many hospitals, stewardship is now considered part of expected clinical practice. Yet despite broad scientific consensus and policy endorsement, stewardship programs remain surprisingly fragile: understaffed, inconsistently funded, and highly sensitive to competing institutional priorities (2, 3).

This tension, between stewardship's demonstrated effectiveness and its operational vulnerability, cannot be adequately explained by a lack of data or by inadequate clinician engagement. Nor is it primarily a problem of prescribing culture or individual leadership. Rather, the core issue lies in how stewardship is positioned within hospital organizations. In many settings, stewardship is treated as a supplementary or optional service, rather than as a fundamental component of patient safety and quality of care. As a result, its continuity often depends on individual commitment and favorable circumstances rather than on formal institutional protection (4).

This reality contrasts sharply with how stewardship is framed in international policy and professional guidance. The World Health Organization's Global Action Plan on Antimicrobial Resistance identifies optimization of antimicrobial use as a health system responsibility, explicitly linking stewardship to patient safety and public health (5, 6). Professional societies have translated these principles into operational guidance. The Infectious Diseases Society of America (IDSA) and the Society for Healthcare Epidemiology of America (SHEA) define stewardship as a coordinated set of institutional interventions, including prospective audit and feedback and preauthorization of selected antimicrobials (7). Similarly, the Centers for Disease Control and Prevention (CDC) Core Elements framework emphasizes leadership commitment, accountability, pharmacy expertise, action, monitoring, reporting, and education as foundational components of hospital stewardship programs (8). There is broad agreement on what stewardship entails and why it matters.

The empirical evidence supporting stewardship is equally consistent. Systematic reviews and meta-analyses demonstrate improvements in antimicrobial prescribing quality, including increased adherence to guidelines, timely de-escalation, and appropriate duration of therapy (9). Other analyses show reductions in infections and colonization with resistant organisms and *C. difficile*, particularly when stewardship is implemented alongside infection prevention measures (2). Taken together, this literature leaves little doubt regarding stewardship's effectiveness. Its persistent fragility therefore cannot reasonably be attributed to scientific uncertainty.

**Figure 1:**
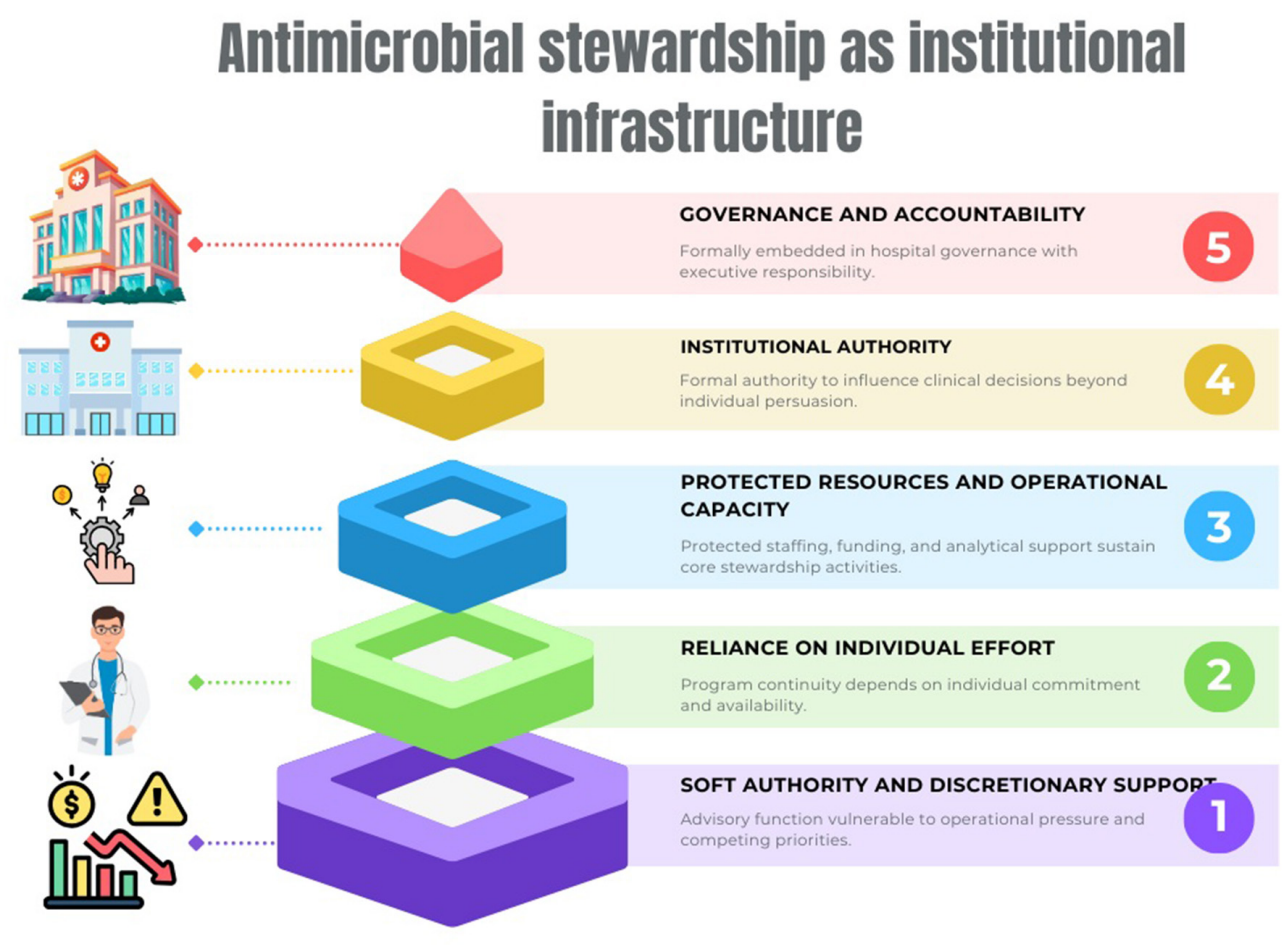
Conceptual representation of antimicrobial stewardship as a continuum of institutional embedding within hospitals. The figure illustrates progression from discretionary, individual-dependent stewardship activities characterized by soft authority to formally embedded patient safety infrastructure supported by defined governance, protected resources, and accountability mechanisms. At the highest level of embedding, stewardship is not only supported by internal hospital governance but also reinforced by external accountability mechanisms, including regulatory expectations, accreditation standards, reimbursement incentives, and public health priorities. The degree of institutional embedding determines the resilience and sustainability of stewardship programs under routine conditions, financial pressure, and organizational stress.

A more productive explanation emerges when attention shifts from individual behavior to organizational structure. Structural fragility refers to formal arrangements, governance frameworks, resource allocation, accountability mechanisms, and incentive structures, that determine whether stewardship is protected or expendable. Stewardship programs may be led by committed professionals and supported by well-designed processes, yet remain precarious if they lack explicit institutional authorization. Governance, in this context, is distinct from leadership; it reflects the formal rules and decision rights that signal what an organization is prepared to safeguard, particularly under pressure. Figure 1 conceptualizes antimicrobial stewardship along a continuum of institutional embedding, from discretionary, individual-dependent activity to formally governed patient safety infrastructure.

Discussions of stewardship often conflate structural and cultural explanations. Prescribing norms and professional attitudes undoubtedly influence antimicrobial use, but culture does not exist independently of structure. Organizational context shapes, reinforces, and constrains behavior. When stewardship activities are formally supported, resourced, and endowed with recognized authority, stewardship-aligned practices are more likely to persist. When stewardship is optional, under-resourced, or easily overridden, even strong professional commitment erodes during periods of operational strain.

One of the clearest manifestations of structural fragility is the configuration of stewardship capacity in many hospitals. Programs are frequently built around a very small number of highly committed individuals, most commonly an infectious diseases physician and a clinical pharmacist, with limited protected time and minimal analytical or informatics support. This model creates obvious vulnerabilities. Absence, redeployment, or staff turnover can rapidly curtail core stewardship activities such as prospective audit and feedback, review of empiric therapy, and support for de-escalation. This dependence on individual effort persists despite repeated calls for defined workforce standards and sustainable funding models (10). It reflects an implicit organizational choice to resource stewardship as peripheral while expecting it to influence prescribing across the entire institution.

A second source of fragility lies in stewardship's limited formal authority. In many hospitals, stewardship teams advise rather than decide. Their influence rests largely on persuasion rather than governance-backed mandates. Under stable conditions, this may suffice. Under pressure, when diagnostic uncertainty, time constraints, and competing priorities dominate clinical decision-making, soft authority is easily sidelined. Concerns are often raised that greater stewardship authority could threaten prescriber autonomy or impose undue rigidity. These concerns merit discussion, but they do not justify the absence of formal institutional authority. Stewardship authority need not be punitive; it can be exercised through clearly defined expectations, escalation pathways, and explicit recognition that stewardship recommendations carry institutional weight.

A third contributor to fragility is the misalignment between stewardship's value and hospital incentive structures. Many of stewardship's benefits are preventive and accrue over time, including fewer resistant infections, fewer adverse drug events, and preservation of antimicrobial effectiveness. While economic analyses at the system level demonstrate substantial returns on investment from stewardship and infection control, these benefits are poorly captured by short-term hospital financial metrics (11). Consequently, stewardship funding is often discretionary and among the first to be reduced during fiscal constraint. Unlike other safety-critical functions, stewardship is not consistently protected by regulation or external accountability mechanisms.

The experience of the United States illustrates how external accountability can influence the institutional position of stewardship. In this setting, antimicrobial stewardship expectations have been incorporated into regulatory and accreditation frameworks, including federal participation requirements and hospital accreditation standards. Such mechanisms can shift stewardship from a locally discretionary activity toward a condition linked to quality oversight, reimbursement, and institutional legitimacy. However, this example also shows that external requirements alone are insufficient if scope, intensity, and implementation quality remain dependent on shifting public health priorities, funding arrangements, and local organizational capacity.

Although much of this discussion reflects experience in high-income settings, similar patterns are observed elsewhere. In low- and middle-income countries, stewardship fragility may arise through different mechanisms, such as limited diagnostic capacity or reliance on externally funded initiatives that sit outside routine hospital governance. In these contexts, stewardship may function effectively while external support persists but becomes difficult to sustain once funding ends. Again, the issue is not the validity of stewardship principles, but their institutional embedding.

The COVID-19 pandemic brought these vulnerabilities into sharp focus. Early in the pandemic, antimicrobial prescribing increased in many hospitals despite low rates of confirmed bacterial co-infection, raising concerns about downstream consequences for AMR (12). At the same time, stewardship activities were widely disrupted. Staff were redeployed, routine audit and feedback were suspended, and priorities shifted rapidly in response to crisis demands (13, 14). Qualitative studies described significant professional strain among stewardship teams, underscoring the close link between program resilience and workforce resilience (15). The pandemic did not create stewardship fragility; it functioned as a stress test, exposing pre-existing structural weaknesses.

Paradoxically, the pandemic also highlighted stewardship's broader value. Stewardship teams contributed to rapid guideline development, antimicrobial access management, treatment standardization, and real-time decision support under uncertainty (16). These activities extend well beyond routine optimization of prescribing and demonstrate that stewardship operates as dual-use infrastructure, supporting both everyday care and crisis response.

If stewardship fragility is structurally produced, it must be addressed through structural solutions. Antimicrobial stewardship should be explicitly recognized as core patient safety infrastructure, supported by protected resources and clear accountability rather than discretionary support. Making organizational support mandatory requires more than general institutional endorsement. At a minimum, hospitals should formally institutionalize a multidisciplinary stewardship structure led by infectious diseases and pharmacy expertise, with protected and compensated time for core team members; administrative support for continuous measurement of antimicrobial use, resistance patterns, and program outcomes; and integration of stewardship activities within quality improvement, patient safety, infection prevention, and pharmacy and therapeutics governance. Core proactive strategies, particularly prospective audit with feedback and preauthorization or formulary restriction for selected antimicrobials, should be embedded as routine institutional processes rather than dependent on individual availability or informal persuasion. In addition, stewardship programs require enabling infrastructure, including electronic health record support, data-reporting capacity, physician champions, and visible hospital leadership commitment. These elements are consistently described in stewardship guidance and implementation studies as central facilitators of effective and sustainable programs, and they provide a practical basis for converting organizational support from discretionary goodwill into a measurable institutional obligation (17, 18).

Regulatory recognition of stewardship programs as mandatory safety functions—analogous to infection prevention—would be one important step, accompanied by defined expectations for staffing, reporting, and executive oversight. Governance should include board-level visibility of antimicrobial use and resistance indicators, embedding stewardship within institutional risk management. Protected budget lines and minimum staffing standards, scaled to hospital size and complexity, would reduce reliance on individual champions and mitigate single-point-of-failure risks. Integration of stewardship principles into electronic prescribing systems and clinical pathways would further support appropriate antimicrobial use by default.

Finally, stewardship capacity should be considered an element of emergency preparedness (18). The pandemic demonstrated that stewardship systems support both routine care and organizational resilience during crises. Governance arrangements should therefore evaluate stewardship not only by its day-to-day performance, but also by its ability to withstand disruption while maintaining core functions (19). Antimicrobial stewardship programs remain among the most effective tools available to hospitals to address antimicrobial resistance. Their persistent fragility does not reflect inadequate evidence, professional resistance, or poor implementation. It reflects governance and resourcing choices that render stewardship optional rather than protected. Continuing to treat antimicrobial stewardship as discretionary is no longer defensible. Without structural embedding as a core organizational function, its benefits remain contingent and easily reversible, to the detriment of both current patient care and future therapeutic options.
